# Identification of unique reciprocal and non reciprocal cross packaging relationships between HIV-1, HIV-2 and SIV reveals an efficient SIV/HIV-2 lentiviral vector system with highly favourable features for in vivo testing and clinical usage

**DOI:** 10.1186/1742-4690-2-55

**Published:** 2005-09-16

**Authors:** Padraig M Strappe, David W Hampton, Douglas Brown, Begona Cachon-Gonzalez, Maeve Caldwell, James W Fawcett, Andrew ML Lever

**Affiliations:** 1 Department of Medicine, University of Cambridge Addenbrooke's Hospital Cambridge CB2 2QQ, UK; 2 Centre for Brain Repair, University of Cambridge, Addenbrooke's Hospital, Cambridge, CB2 2QQ, UK

## Abstract

**Background:**

Lentiviral vectors have shown immense promise as vehicles for gene delivery to non-dividing cells particularly to cells of the central nervous system (CNS). Improvements in the biosafety of viral vectors are paramount as lentiviral vectors move into human clinical trials. This study investigates the packaging relationship between gene transfer (vector) and Gag-Pol expression constructs of HIV-1, HIV-2 and SIV. Cross-packaged vectors expressing GFP were assessed for RNA packaging, viral vector titre and their ability to transduce rat primary glial cell cultures and human neural stem cells.

**Results:**

HIV-1 Gag-Pol demonstrated the ability to cross package both HIV-2 and SIV gene transfer vectors. However both HIV-2 and SIV Gag-Pol showed a reduced ability to package HIV-1 vector RNA with no significant gene transfer to target cells. An unexpected packaging relationship was found to exist between HIV-2 and SIV with SIV Gag-Pol able to package HIV-2 vector RNA and transduce dividing SV2T cells and CNS cell cultures with an efficiency equivalent to the homologous HIV-1 vector however HIV-2 was unable to deliver SIV based vectors.

**Conclusion:**

This new non-reciprocal cross packaging relationship between SIV and HIV-2 provides a novel way of significantly increasing bio-safety with a reduced sequence homology between the HIV-2 gene transfer vector and the SIV Gag-Pol construct thus ensuring that vector RNA packaging is unidirectional.

## Background

Viral vectors based on primate and non-primate lentiviruses have been shown to be efficient for gene delivery to a variety of cell types both *in vitro *and *in vivo *and may offer considerable advantages in gene therapy strategies [1,2]. Lentiviral vectors can provide stable gene expression following integration into the host chromosome and pseudotyping of these vectors with heterologous envelopes such as the G protein of Vesicular stomatitis virus (VSV) has provided a broad cell tropism [3]. Lentiviral vectors are particularly suited for transduction of non-dividing cells [4] such as those of the central nervous system [5] exemplified by successful therapeutic gene transfer to the brain of primates for treatment of experimentally induced Parkinson's disease [6]. Packaging of unspliced vector mRNA in the producer cell line is a key part in process of lentiviral vector production and measures to increase the packaging efficiency and to reduce self packaging of the Gag-Pol or other helper construct have contributed to increased vector titre and biosafety [7]. Lentiviral RNA packaging is achieved by an interaction between an RNA structure known as the packaging signal or psi and the nucleocapsid (NC) domain of the Gag structural polyprotein. This highly specific process results in the selection of unspliced viral mRNA from a high background of cellular mRNA. The packaging signals of several lentiviruses have been mapped by deletion and mutational analysis. For HIV-1, sequences between the major splice donor and the start codon of Gag have been shown to be important for efficient packaging [8]. HIV-1 may be the exception amongst lentiviruses since for HIV-2 and SIV, sequences upstream of the splice donor predominantly contribute to mRNA packaging [9,10] and in FIV regions in U5 and in the Gag coding sequence appear to be the major signals [11,12]. RNA packaging in HIV-2 has been shown to involve two novel mechanisms to increase specificity, cotranslational packaging and competition for limiting Gag polyprotein [13]. These differences in the location of the major packaging determinants may contribute to the ability of viral mRNA to be cross packaged by a heterologous Gag protein. The localisation of RNA capture in the cell is unclear although recent evidence suggests that the centrosome may be the primary site [14] and that the psi signal may act as a subcellular localisatio signal as well as a high affinity binding site for Gag. The resulting RNA-protein complex is then targeted to the plasma membrane where virion budding takes place.

The ability of one lentiviral Gag to cross-package the unspliced mRNA of another lentivirus species has been well demonstrated for HIV-1, which can cross-package HIV-2 [15], SIV [16,17] and FIV [18]. Both SIV and FIV Gag-Pol have been shown to cross-package HIV-1 mRNA [16,18], however HIV-2 Gag-Pol is unable to package HIV-1 mRNA [15]. How closely this reduced efficiency correlates with the effectiveness of gene transfer of cross-packaged vectors has not been assessed, in particular in appropriate primary cells. Cross-packaged lentiviral vectors have been shown to infect predominantly dividing cells in culture but transduction of neurons and CD34+ lymphocytes has only been shown qualitatively [16]. However chimeric vectors based on an SIV genome and an HIV-1 core were unable to transduce dendritic cells and had a reduced ability to transduce primary macrophages [19].

The production of lentiviral vectors for clinical trials requires that preparations do not contain replication competent lentiviruses (RCL). Development of PCR and sensitive culture based methods for detection of RCLs have confirmed the absence of RCLs in large production lots [20,21]. Production of RCLs can occur through homologous recombination, thus limiting the sequence similarity between the Gag-Pol construct and gene transfer vector will reduce the possibility of a recombination event. Gag-Pol and gene transfer vectors based on different lentiviruses will significantly reduce the risk of RCL production.

Transduction of the cells of the central nervous system (CNS), both brain and spinal cord, with lentiviral vectors has been well documented and long term therapeutic transgene expression has been reported with only a low level or transient immune/inflammatory response [22,23]. Furthermore, transduction of neural stem cells with lentiviral and adeno associated viral vectors expressing therapeutic genes that will affect differentiation and serve as markers of cell fate is a promising approach for procuring cells for transplantation into degenerated or damaged areas of the brain. Such cells have the potential to be useful for the treatment of Parkinson's disease, spinal cord injury and other inflammatory or destructive conditions of the CNS[24,25].

We investigated the cross packaging ability of the Gag-Pol components of HIV-1, HIV-2 and SIV and found a unique non-reciprocal packaging relationship between SIV Gag-pol and vectors based on HIV-2.

In this paper the tropism of these viruses is quantitated by examining the ability of a series of cross-packaged lentiviral vectors based on HIV-1, HIV-2 and SIV to transduce primary mixed glial cells which, are the predominant cell type in the injured brain or spinal cord. Qualitative data is also presented on the transduction of primary neuronal embryonic stem cells with cross-packaged vectors.

## Results

### Cross-Packaging of lentiviral RNA

**Figure 3:**
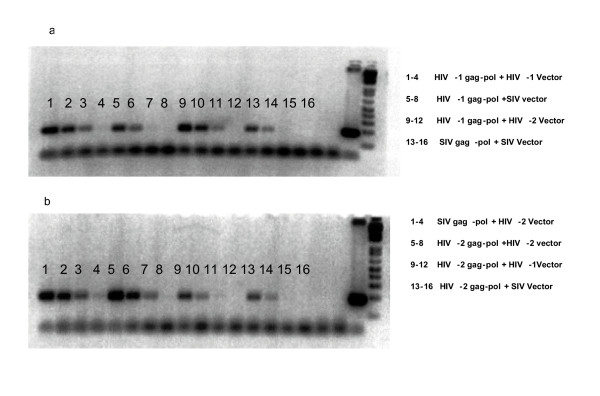
Limiting dilution RT PCR of Virion associated GFP RNA. For each viral vector, four PCR s were performed containing a target cDNA at neat, 1/10, 1/20 and 1/40 dilution. **A**: Lanes 1–4, HIV-1 Gag-pol + HIV-1 Vector, Lanes 5–8, HIV-1 Gag-pol + SIV vector, Lanes 9–12, HIV-1 Gag-pol + HIV-2 vector, Lanes 13–16, SIV Gag-pol + SIV vector. **B**: Lanes 1–4, SIV Gag-pol + HIV-2 vector, Lanes 5–8, HIV-2 Gag-pol + HIV-2 vector, Lanes 9–12, HIV-2 Gag-pol + HIV-1 vector, Lanes 13–16, HIV-2 Gag-pol + SIV vector.

Following concentration of viral vectors by ultracentrifugation, viral vector particle number was assessed by the reverse transcriptase assay, which gives a quantitative measure of RT in ng. The concentration of each viral vector was normalised to 4 ng/ μl following previous optimisation. The level of vector RNA in the producer cells was comparable as judged by fluorescence of the cells caused by expression of the transfected GFP containing vector. The levels of RNA packaged in virions were assessed by RT-PCR of the packaged transgene GFP, using specific primers. Figure 3A and 3B shows a limiting dilution PCR analysis of virion extracted RNA, reverse transcribed to cDNA and diluted serially from 1/10 and 1/20 to 1/40. Electrophoresis of PCR products reveals a limit of positivity and signal strength. In Figure 3(A) HIV-1 Gag-Pol is seen to efficiently package HIV-1 RNA and can also cross package HIV-2 vector RNA at similar levels, both to a limiting dilution of 1/20. In comparison cross packaging of SIV vector RNA by HIV-1 Gag-Pol is reduced and is similar to levels of SIV vector RNA packaged by SIV Gag-Pol to only a limiting dilution of 1/10. In Figure 3(B), SIV Gag-Pol efficiently cross packages HIV-2 vector RNA to a limiting dilution of 1/40, which is greater than the SIV homologous vector system (1/10) and the SIV Gag-pol + HIV-GFP vector system (1/10, data not shown). The ability of HIV-2 Gag-Pol to cross package HIV-1 and SIV vector RNA is significantly reduced compared to the homologous HIV-2 system which showed similar levels of packaged RNA to the HIV-1 homologous vector system.

### Gene transfer efficiency of cross packaged vectors

**Figure 4:**
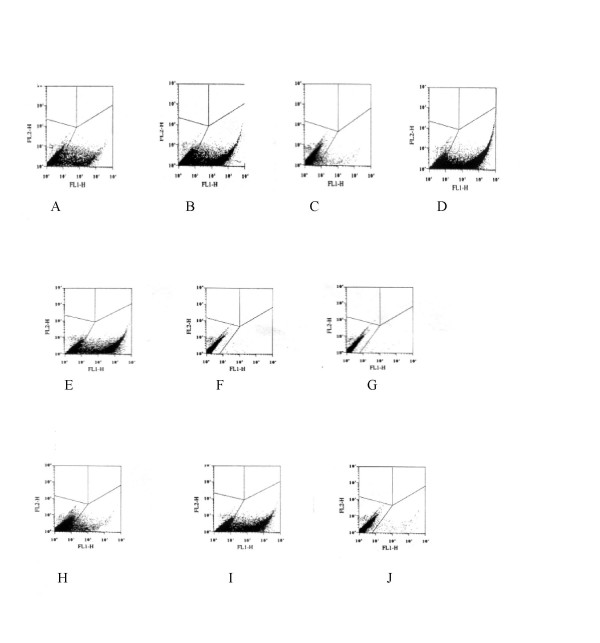
FACS analysis of GFP expression in SV2 cells transduced with homologous and cross-packaged lentiviral vectors (10 ng of vector). Lower Right hand quadrant represents GFP positive cells. HIV-1 Gag-Pol + HIV-1GFP vector (A), HIV-1 Gag-Pol + HIV-1 cPPT-GFP vector (B), HIV-1 Gag-Pol + SIV GFP vector (C), HIV-1 Gag-Pol + HIV-2 GFP vector (D). HIV-2 Gag-Pol + HIV-2 GFP vector (E), HIV-2 Gag-Pol + SIV GFP vector (F), HIV-2 Gag-Pol + HIV-1 GFP vector (G). SIV Gag-Pol + SIV GFP vector (H), SIV Gag-Pol + HIV-2 GFP vector (I), SIV Gag-Pol + HIV-1 GFP vector (J).

The semi quantitative PCR approach demonstrates levels of vector RNA packaged in comparable concentrations of virions, however the assay does not reflect the gene transfer efficiency of cross-packaged vectors. To address this, SVC2 cells were transduced with a range of vector-virion preparations at differing concentrations as measured by RT-assay. Figure 4 shows a series of FACS plots of GFP positive cells (lower right quadrant) following transduction with viral vector and this data is also described in tables 4A to 4C. HIV-1 Gag-Pol was used to package two separate HIV-1 vectors (+/-cPPT sequence), the gene transfer vector containing the cPPT demonstrated an increased transduction rate of SVC2 cells up to almost two fold with an input viral vector of 10 ng. Transfer of 20 ng of an HIV-2 vector packaged by HIV-1 Gag-Pol showed a similar transduction efficiency to that of the HIV-1 cPPT vector packaged by HIV-1 Gag-Pol, suggesting that the HIV-2 cPPT region also contributed to increased transduction. Transfer of an SIV vector expressing GFP, cross-packaged by HIV-1 Gag-Pol was significantly lower, almost six fold, compared to the homologous HIV-1 viral vector (-cPPT). It is not certain why this is nor why the homologous SIV system gave low/poorly reproducible results. Vector expression appeared comparable in producer cells. SIV Gag-Pol cross packaged and transferred an HIV-2 GFP vector at levels slightly higher than the homologous HIV-1 vector system. This is in contrast to the lack of gene transfer of a HIV-1 vector packaged by SIV Gag-Pol. The levels of HIV-2 vector RNA packaged by SIV Gag-Pol (Figure 3B) are also reflected in the high gene transfer efficiency. This packaging relationship between SIV and HIV-2 would appear to be non-reciprocal, with lower amounts of SIV vector RNA packaged by the HIV-2 Gag-Pol (Figure 3B) and no evidence of any significant gene transfer. Comparing the HIV-1 and HIV-2 homologous vector systems showed that levels of gene transfer to SVC2 cells were slightly higher for HIV-2 compared to a cPPT negative HIV-1 vector but lower when compared to the HIV-1 vector containing the cPPT region. HIV-2 Gag-Pol would appear to have no ability to cross-package and transfer HIV-1 vector, which is similar to a previous study [15] with no significant transduction of SVC2 cells.

**Figure 6:**
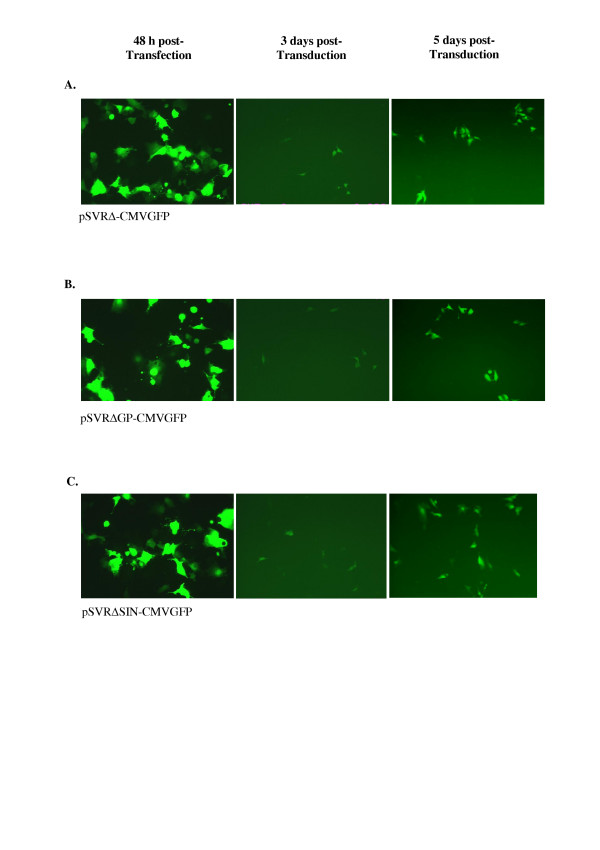
GFP expression from HIV-2 vectors following transfection inot produced cells and in cells transduced with the packaged vectors.

One obvious difference between the vectors packaged is the presence of considerably more potential *cis *acting sequence in the HIV-2 based vector compared to the HIV-1 and SIV vectors. It is conceivable that the presence of extended *cis *acting sequence in the *gag *and *pol *genes alters the efficiency of packaging. From data using HIV-1 based vectors this would seem to be unlikely since the minimal HIV based vector packages at least as well as a less fully deleted version. Nevertheless to establish closer comparability we generated a series of further deletions in the HIV-2 based vector and compared gene transfer efficiency to that achieved with the minimally deleted vector. The vector series included one with near complete deletion of the Gag/Pol coding regions (pSVRΔGP-CMVGFP) and also the generation of a self inactivating (SIN) vector (pSVRΔSIN-CMVGFP) created by additional deletion in the 3' UTR. This will be copied into the 5'LTR during reverse transcription and thus inactivate the 5'LTR promoter such that expression of the transgene depends on the internal promoter. The deletion removing the Gag-Pol region extends into the first coding exons of Tat and Rev thus both of these vectors will be defective for these regulatory proteins and they are closely comparable to the HIV-1 and SIV vectors used. Using these HIV-2 based constructs we were able to demonstrate no difference in gene transfer ability with either the more extensively deleted or the SIN mutated vector. Examples of gene transfer efficiency are shown in Figure 6 in which the level of GFP expression on transfection and transduction of all of the HIV-2 vectors is comparable.

### Transduction of CNS cell types

**Figure 7:**
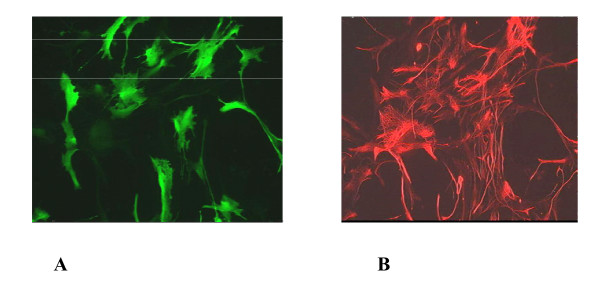
Transduction of rat mixed glial cells with a HIV-2 based lentiviral vector packaged by SIV gag-pol. (A) GFP expression in lentivector transduced cells. (B) GFAP co-staining of astrocytes.

**Figure 8:**
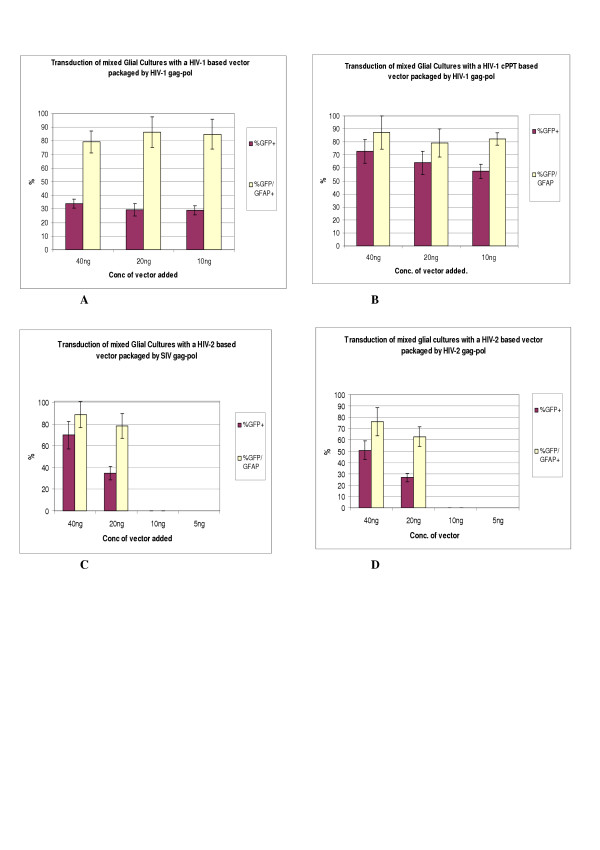
Transduction of Rat primary mixed glial cultures with Lentiviral vectors based on HIV-1 packaged by HIV-1 Gag-pol(A), HIV-1 +cPPT vector packaged by HIV-1 Gag-pol (B), HIV-2 vector packaged by SIV Gag-pol (C) and HIV-2 vector packaged by HIV-2 Gag-pol (D). Error bars indicate within experimental SEM.

We decided to verify this unreported cross-packaging and gene transfer relationship between SIV Gag-Pol and a HIV-2 vector by first transducing rat primary mixed glial cultures. The cultures were transduced with either 40 ng or 20 ng of viral vector and the efficiency of transduction compared to that achieved with HIV-1 and HIV-2 homologous vector systems. Cells were immunostained for GFP expression and the astrocyte marker GFAP (Figure 7), and counted (Figure 8). Transducing the glial cultures with 20 ng of a SIV gag-pol+HIV-2 GFP viral vector resulted in GFP positivity in over 30% cells and approximately 80% of these positive cells were astrocytes. A similar transduction rate was seen with the HIV-1 homologous vector system, which lacks the cPPT sequence using 20 ng of viral vector. At the same viral vector concentration, the HIV-2 homologous vector system transduced approximately 25% of glial cells with 62% of these cells staining for GFAP. The effect of the cPPT sequence on HIV-1 viral vector transduction is evident with over 60% of glial cell expressing GFP with 20 ng of input vector and approximately 58% with 10 ng of vector. In summary, the gene transfer efficiency of the HIV-2 GFP vector to be cross packaged by SIV Gag-pol to glial cells was similar to both the HIV-1 and HIV-2 homologous vector systems.

**Figure 9:**
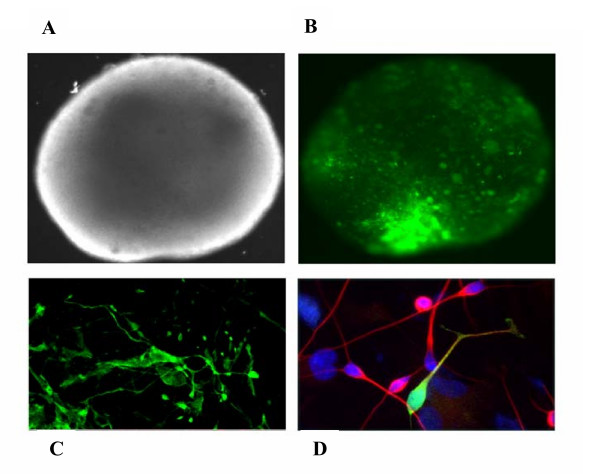
Transduction of neural stem cells by a HIV-2 based GFP lentiviral vector packaged by SIV-2 Gag-Pol. (A) Phase contrast image through growing neurosphere (upper left). (B) Fluorescent image of neurosphere in A expressing GFP 72 hours post transduction (upper right). (C) Confocal image through neurosphere expressing GFP (lower left) (D) Neurons derived from human neurosphere 7 days post differentiation (lower right). Red represents β tubulin III, green – GFP, Hoechst stain (blue) nuclei. Arrow denotes double labelled cell. Magnification in A and B = 10×, in C = 100×, D = 40×

Transduction of human embryonic neuronal stem cells was also performed using the HIV-1 and HIV-2 homologous vector system (not shown) and with the SIV Gag-Pol /HIV-2 GFP. The transduction efficiency was assessed qualitatively by fluorescence microscopy using 20 ng of viral vector, and Figure 9 shows that the SIV Gag-pol/HIV-2 GFP cross packaged vector system transduced both astrocytes and neurons post differentiation as demonstrated by immunostaining with GFAP (astrocytes) and beta-tubulin (neurons). The cross packaged vector system performed as well as the HIV-1 and HIV-2 homologous vector systems with astrocytes being transduced at a slightly higher efficiency.

## Discussion

Both lentiviruses and other retroviruses have shown an ability to cross package other viral genomes with HIV-1 Gag-Pol demonstrating the greatest cross packaging ability. Non-reciprocal packaging relationships such as have been demonstrated in HIV-1 and HIV-2 [15] or spleen necrosis virus and murine leukaemia virus [26] suggest that individual viruses have different packaging mechanisms relating possibly to the availability of the Gag protein or the position of the RNA packaging signal relative to the major splice donor or other as yet unknown factors. In this study we demonstrate for the first time a non-reciprocal packaging relationship between SIV and HIV-2. Interestingly, the major packaging determinant of both HIV-2 and SIV has been shown to be upstream of the major splice donor [9,10] and by inference one would expect SIV to demonstrate the same co-translational packaging process as HIV-2 [13]. SIV Gag-Pol has been previously reported to cross package HIV-1 and FIV unspliced vector mRNA [16,7,18] however the gene transfer ability of these chimeric vectors has been limited. We could not demonstrate any appreciable gene transfer of an HIV-1 based vector cross-packaged by SIV Gag-Pol, which is in contrast to a previous study [16], where transduction of both dividing and non-dividing cells was demonstrated. Nor was gene transfer of the HIV-1 GFP seen when packaged by HIV-2 Gag-Pol, in contrast to a previous report [15].

**Figure 5:**
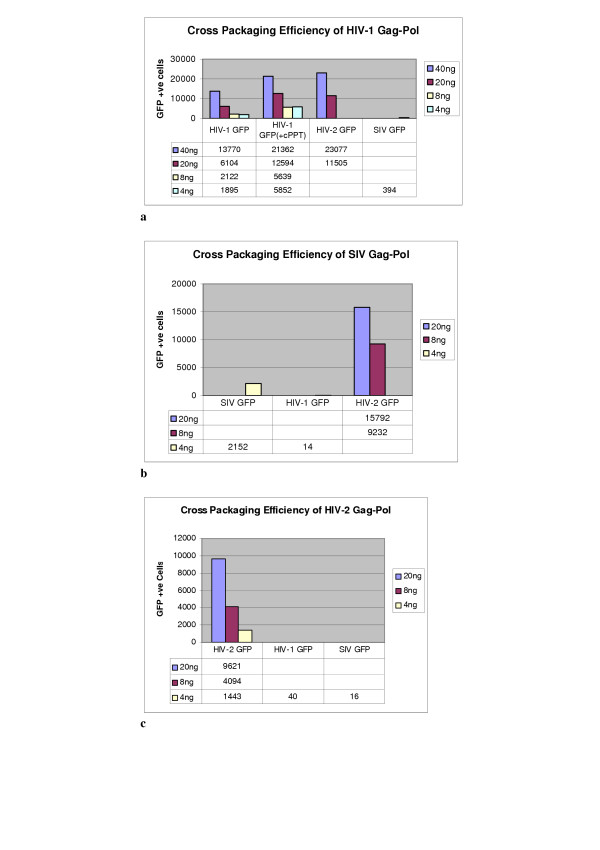
**a **Quantitative assessment of GFP transfer to SVC2 cells by FACS analysis using HIV-1 Gag-Pol to package gene transfer vectors based on HIV-1 (+/- cPPT sequence), HIV-2 and SIV. A range of Viral vector concentrations from 40 ng to 4 ng of Reverse Transcriptase was used. (Blank = No data). **b **Quantitative assessment of GFP transfer to SVC2 cells by FACS analysis using SIV Gag-Pol to package gene transfer vectors based on SIV, HIV-1 and HIV-2. A range of Viral vector concentrations from 20 ng to 4 ng of Reverse Transcriptase was used. **c **Quantitative assessment of GFP transfer to SVC2 cells by FACS analysis using HIV-2 Gag-Pol to package gene transfer vectors based on, HIV-2, HIV-2 and SIV. A range of Viral vector concentrations from 20 ng to 4 ng of Reverse Transcriptase was used.

SIV Gag-Pol packaged similar levels of HIV-1 RNA compared to the homologous SIV vector system (Figure 3A and 3B), however a significant decrease in gene transfer was demonstrated with the SIV Gag-Pol/HIV-1 GFP vector when 4 ng of vector was used to transduce SVC2 cells (Figure 5B). A similar observation was demonstrated with HIV-2 Gag-Pol, which packaged equal levels of HIV-1 GFP and SIV GFP vector RNA and showed no appreciable gene transfer with 4 ng of vector. The RT-PCR data on virion extracted RNA suggests that low levels of RNA are being packaged. Why this does not translate into detectable gene transfer is not clear although the RT-PCR does not reveal whether complete or damaged genomes are being packaged. Gene transfer may be a threshold phenomenon in which many virions contain defective genomes and only a few have a full genomic RNA. Alternatively there may be an additional block in functional gene transfer either at reverse transcription or integration. Indeed, there is no reported data on the function of SIV reverse transcriptase or integrase in an HIV-1 backbone. The cross packaging ability of HIV-1 Gag-Pol was demonstrated by its ability to package both HIV-2 and SIV RNA and effect GFP gene transfer. HIV-1 Gag-Pol packaged a greater level of HIV-2 RNA than SIV RNA and a significantly greater number of cells were transduced with the HIV-1 Gag-Pol/HIV-2 GFP vector.

One advantage of a chimeric lentiviral vector is a reduction in the risk of development of a replication competent retrovirus which may occur through a recombination event due to sequence homology between the Gag-Pol and gene transfer constructs. However it is important to assess the gene transfer capabilities of these chimeric vectors in suitable primary cells. This has been highlighted in a study where a gene transfer vector based on SIV packaged by HIV-1 Gag-Pol showed a reduced transduction efficiency of human dendritic cells associated with a post-entry defect. [19]. A second major advantage of this chimeric system is the ability to deliver a cross-packaged vector to a simian animal model with a vector based on SIV Gag-Pol packaging an HIV-2 genome. The same combination could subsequently be used in humans allowing biosafety and bio-distribution studies to be performed directly without the necessity for surrogate systems. This is not possible with an HIV-1 based system and would give the SIV/HIV-2 system considerable advantages over other primate lentiviral combinations.

Rat astrocytes are the major cell type associated with the glial scar resulting from injury to the CNS [27] and human fetal embryonic neural stem cells offer the potential for regenerating damaged areas of the CNS [28]. Engraftment of neural stem cells transduced with a lentiviral vector based on HIV-1 has been demonstrated with a high level and duration of transgene expression[29]. Our results demonstrate that both the HIV-1 and HIV-2 homologous GFP lentivectors efficiently transduced rat primary astrocytes. Similar to previous studies on the effect of the cPPT sequence on gene transfer [30,31] our data shows that the presence of the cPPT sequence in the HIV-1 vector results in a two fold increase in transduction efficiency, similar to the HIV-2 homologous vector system which also contains the HIV-2 cPPT in the *pol *sequence. The SIV Gag-Pol/ HIV-2 GFP vector also transduced primary astrocytes with efficiency similar to the HIV-1 cPPT homologous vector system, indicating no associated post-entry defects. Efficient transduction of human fetal embryonic neural stem cells was also shown with the cross packaged SIV Gag-Pol/HIV-2 GFP vector highlighting the ability of this vector to transduce human cells.

## Conclusion

We have identified a non reciprocal cross packaging relationship between SIV Gag-Pol and a HIV-2 based GFP vector, which demonstrated equivalent transduction efficiencies in 293T cells, rat primary astrocytes and embryonic stem cells as that of homologous HIV-1 and HIV-2 vector systems. The efficiency of the combination correlates with the level of vector RNA packaged indicating that this is a major determinant of vector efficiency. It suggests that there are as yet unidentified differences in the RNA capture mechanisms of HIV-1, HIV-2 and SIV.

The implications for testing of lentiviral vector biosafety are potentially very important. Testing in appropriate animal models is a major concern associated with the use of lentiviral vectors in clinical trials. As HIV-1 only causes AIDS in humans, there is presently no animal model to test the safety of HIV-1 based vectors. However animal models based on Asian macaques and baboons exist for SIV and HIV-2, respectively which may be applicable to testing the biosafety of SIV cross packaged HIV-2 lentiviral vectors.

## Methods

### Lentiviral vectors

**Figure 1:**
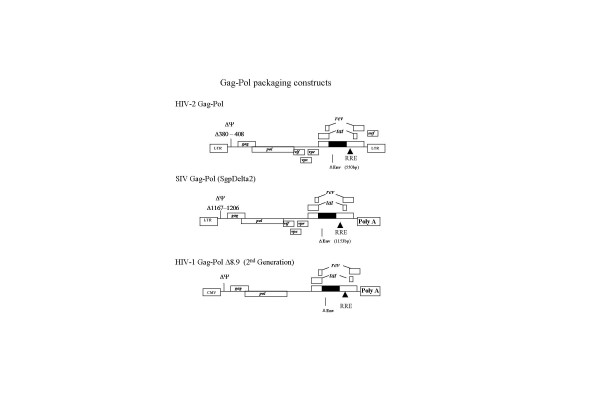
Gag-Pol packaging constructs.

**Figure 2:**
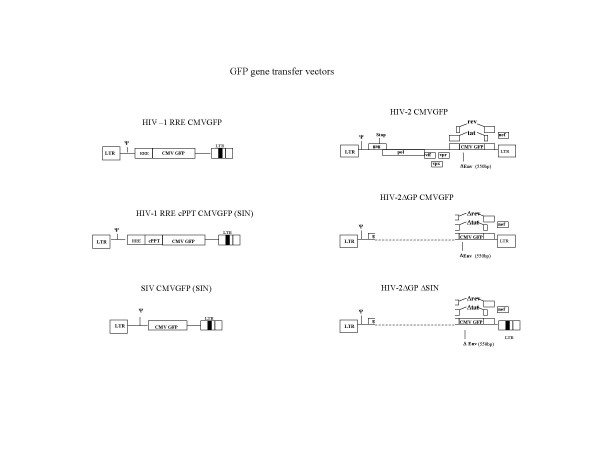
GFP gene transfer vectors. The dotted line indicates a deletion

The lentiviral gene transfer vectors and Gag-Pol expression constructs are outlined in Figures 1 and 2. The constructs based on HIV-1 and SIV have been previously described [4,32] and were kind gifts of D. Trono and K. Uberla. The HIV-1 gene transfer vector HR'GFP was modified to include the HIV-1 central polypurine (cPPT) tract or DNA flap sequence. The sequence was PCR amplified and cloned into the unique Cla1 site upstream of the Rev Responsive Element (RRE) sequence using cPPT primer sequences described [30]. The HIV-2 gene transfer vector was also modified from the construct pSVRΔNBPuroΔH [13] by replacing the SV40-Puromycin construct with a CMV-GFP reporter gene construct to create pSVRΔ-CMVGFP. The HIV-2 Gag-Pol construct, (pSVRΔNBDM) contains a deletion in the 5'untranslated region, which has been shown to abrogate packaging [13].

### Construction of minimal HIV-2 based vectors

pSVRΔNBΔH [13] was digested with *BsmBI*, and a *ClaI *linker was inserted into the site. *ClaI *and *EcoRV *digestion of this produced two DNA fragments, the smaller of which (nucleotides 1101–6128 encompassing *gag *and *pol *sequences) was discarded. The remaining fragment was religated and formed pSVRΔGP. CMVGFP was obtained from *SalI *digestion of pSVRΔ-CMVGFP and ligated into the *SalI *linker of dephosphorylated pSVRΔGP to give pSVRΔGP-CMVGFP.

The HIV-2 U3 region contains a TATA box, core enhancer regions, and Sp1, κB and peri-κB binding sites that are responsible for transcription from the 5'LTR. This 141 bp region (nucleotides 9329–9470) was deleted in the 3'LTR to produce a SIN vector as follows. The 3'LTR was removed from pSVRΔGP, by *BamHI *and *XbaI *digestion, and subcloned into pBluescript II KS. Site directed mutagenesis introduced *BglII *restriction sites at the 5' and 3' ends of the 141 bp region that was to be deleted. Mutagenesis was carried out as in two stages using the following primers: stage 1, upstream mutagenesis:- 5'-GGAATACCATTTAGTTAAAGATCTGAACAGCTATACTTGGTCAGGG-3' and :- 5'-CCCTGA CCAAGTATAGCTGTTCAGATCTTTAACTAAATGGTATTCC-3'; for stage 2, downstream mutagenesis, 5'-CGCCCTCATATTCTCTGTATAGATCTACCCGCTAGCTTGCATTG-3' and 5'-CAATGCAAGCTAGCGGGTAGATCTATACAGAGAATATGAGGGCG-3'.

The 141 bp U3 region was removed from the plasmid by *BglII *digestion and the plasmid religated. *BamHI *and *XbaI *digestion of the plasmid and religation of the Δ3'LTR into pSVRΔGP created the pSVRΔSIN vector. CMVGFP was inserted as described to produce the vector pSVRΔSIN-GFP

### Lentiviral vector production

Lentiviral vectors were produced by calcium phosphate transfection of 293T cells grown in DMEM media and 10% FCS with 7 μg of the gene transfer vector, 7 μg of the Gag-Pol construct, 3 μg of a Rev expressor and 3 μg of the VSV-G heterologous envelope. For HIV-2 and SIV vector production the Rev expressor was omitted. 24 hours following transfection the media was replaced and supernatant containing recombinant virions was recovered 48 hours post transfection. Virions were concentrated by ultracentrifugation for 2.5 hours at 25,000 RPM in an SW28 Beckmann rotor. The viral pellet was resuspended in 300 μl of tissue culture media, aliquoted and stored at -70°C.

Lentiviral vectors were quantitated using a commercially available RT-assay (Cavid Tech, Uppsala) Vector preparations were measured in duplicate and normalised to a concentration of 8 ng of RT per μl. Although the sensitivity of the assay for different RTs may be slightly different the fact that each Gag-Pol construct is being used to package each vector provides an internal control.

Levels of RNA packaging were assessed by RT-PCR of Virion associated RNA. Virion RNA was extracted using the Qiagen Viramp kit from 10 ng of virus (RT levels). Following extraction the RNA was also treated with RNase Free DNase for 10 mins at 37°C and the DNase was in activated by incubation at 70°C for a further 10 mins. An aliquot of RNA was reverse transcribed to cDNA using the Promega Improm RT system with an antisense GFP primer (AAGTCGTGCTGCTTCATGTG). The cDNA was then serially diluted and amplified using a sense primer (GACGTAAACGGCCACAAGTT) and the antisense primer. Amplified products were resolved by agarose gel electrophoresis and EtBR staining.

The transduction efficiency of cross-packaged vectors was assessed by FACS analysis of GFP positive cells. A range of viral vector concentration from 40 ng to 4 ng was used to transduce 1 × 10^6 ^of fibroblast SV2C cells in a six well plate. Viral vector was diluted in DMEM containing 6 μg/ml polybrene and cells were exposed to virus for 5 hours. The media was then replaced and GFP expression was assessed at time periods after 72 hours post transduction.

### Glial cell Culture and Stem cell culture

Primary mixed glial cultures were prepared from the brains of newborn rats > 3 days old by dissociation of whole cortex in trypsin, then cultured in poly-D-lysine coated flasks in DMEM/10% FCS. Mixed glial cultures were derived from these cells, once they were confluent, by trypsinisation. The cells were then resuspended in DMEM containing 10% FCS and 1% PSF and centrifuged at 10,000 RPM for 5 minutes. The supernatant was removed and cells were resuspended in DMEM/10% FCS and plated onto Poly-D-Lysine coated coverslips in 24 well plates. Transduction of glial cultures with lentiviral vectors was carried out as described for SV2C cultures. 72 hours post transduction; glial cultures were fixed in 4% paraformaldehyde and stored in PBS at 4°C prior to immunostaining.

Human fetal neuronal stem cell culture was performed as described previously [33]. Transduction of Stem cell cultures of cortical origin was performed with 20 ng of viral vector in DMEM/ HAMS F12 (2:1), 1% N2, EGF (20 ng/ml) FGF-2 (20 ng/ml) and heparin (5 mg/ml) for four hours followed by replacement of the media. Cells were allowed to differentiate on poly-L-lysine/laminin coated coverslips followed by replacement of the media 72 hours post transduction. Cells were fixed in 4% paraformaldehyde after a further 96 hours, followed by immunostaining for GFP, GFAP and β-Tubulin III.

### Immunostaining

Lentiviral vector transduced mixed glial cultures were first blocked using 3% goat serum in TXTBS (0.2% triton X-100, in Tris Buffered Saline) for one hour. Monoclonal anti GFAP (Sigma, 1:500) and polyclonal goat anti rabbit GFP (Molecular Probes), 1: 1000) were diluted in TXTBS with 1% normal goat serum (NGS) for 2 hours. Cells were then washed in TBS for 3 × 10 minutes. Cells were then incubated with secondary antibodies, goat anti mouse Alexa (Molecular Probes, 1:500) and biotinylated goat anti rabbit (Amersham Biosciences, 1:500) for 90 minutes. Following a second 3 × 10 minute wash in TBS, Streptavidin-FITC (Serotec, 1:100) was added in TBS with 1% NGS and Bis-benzamide (Sigma, 1:5000). Coverslips were then mounted in Fluorosave reagent (Calbiochem). Cell counts of immunostained mixed glial cultures were performed from one edge of the coverslip all the way across to the other, horizontally and vertically. A 0.5 mm^2 ^area was counted every 1.5 mm.

## Competing interests

PS, DB and AML are inventors on various patents filed by the University of Cambridge containing usage claims for chimeric lentiviral vectors. There are no licences currently associated with these patents.

## Authors' contributions

PS, AML and JWF jointly conceived of these studies. PS produced and titered the lentiviral vectors, performed FACS analysis and RT-PCR analysis and transduced cell lines, primary glial cells and neural stem cells. DWH produced the primary mixed glial cultures. DB cloned the CMV-GFP cassette into the HIV-2 vector and performed the comparative analysis of the HIV-2 vectors. BCG cloned the HIV-1 cPPT region into the HIV-1 vector. MC produced the neural stem cell cultures. PS drafted this manuscript, which was critically reviewed by AML and JWF.
